# Reduced Risk of *Plasmodium vivax* Malaria in Papua New Guinean Children with Southeast Asian Ovalocytosis in Two Cohorts and a Case-Control Study

**DOI:** 10.1371/journal.pmed.1001305

**Published:** 2012-09-04

**Authors:** Anna Rosanas-Urgell, Enmoore Lin, Laurens Manning, Patricia Rarau, Moses Laman, Nicolas Senn, Brian T. Grimberg, Livingstone Tavul, Danielle I. Stanisic, Leanne J. Robinson, John J. Aponte, Elijah Dabod, John C. Reeder, Peter Siba, Peter A. Zimmerman, Timothy M. E. Davis, Christopher L. King, Pascal Michon, Ivo Mueller

**Affiliations:** 1 PNG Institute of Medical Research, Madang, Papua New Guinea; 2 Department of Medicine & Pharmacology, University of Western Australia, Perth, Australia; 3 Swiss Tropical & Public Health Institute, Basel, Switzerland; 4 Center of Global Health & Diseases (CGHD), Case Western Reserve University, Cleveland, Ohio, United States of America; 5 Infection & Immunity Division, Walter+Eliza Hall Institute, Parkville, Victoria, Australia; 6 Barcelona Centre for International Health Research (CRESIB, Hospital Clínic-Universitat de Barcelona), Barcelona, Spain; 7 The Burnet Institute, Melbourne, Australia; 8 Veterans Affairs Medical Center, Cleveland, Ohio, United States of America; 9 Faculty of Health Sciences, Divine Word University, Madang, Papua New Guinea; KEMRI/Wellcome Trust Research Programme, Kenya

## Abstract

**Background:**

The erythrocyte polymorphism, Southeast Asian ovalocytosis (SAO) (which results from a 27-base pair deletion in the erythrocyte band 3 gene, *SLC4A1Δ27*) protects against cerebral malaria caused by *Plasmodium falciparum*; however, it is unknown whether this polymorphism also protects against *P. vivax* infection and disease.

**Methods and Findings:**

The association between SAO and *P. vivax* infection was examined through genotyping of 1,975 children enrolled in three independent epidemiological studies conducted in the Madang area of Papua New Guinea. SAO was associated with a statistically significant 46% reduction in the incidence of clinical *P. vivax* episodes (adjusted incidence rate ratio [IRR] = 0.54, 95% CI 0.40–0.72, *p*<0.0001) in a cohort of infants aged 3–21 months and a significant 52% reduction in *P. vivax* (blood-stage) reinfection diagnosed by PCR (95% CI 22–71, *p* = 0.003) and 55% by light microscopy (95% CI 13–77, *p* = 0.014), respectively, in a cohort of children aged 5–14 years. SAO was also associated with a reduction in risk of *P. vivax* parasitaemia in children 3–21 months (1,111/µl versus 636/µl, *p* = 0.011) and prevalence of *P. vivax* infections in children 15–21 months (odds ratio [OR] = 0.39, 95% CI 0.23–0.67, *p* = 0.001). In a case-control study of children aged 0.5–10 years, no child with SAO was found among 27 cases with severe *P. vivax* or mixed *P. falciparum*/*P. vivax* malaria (OR = 0, 95% CI 0–1.56, *p* = 0.11). SAO was associated with protection against severe *P. falciparum* malaria (OR = 0.38, 95% CI 0.15–0.87, *p* = 0.014) but no effect was seen on either the risk of acquiring blood-stage infections or uncomplicated episodes with *P. falciparum*. Although Duffy antigen receptor expression and function were not affected on SAO erythrocytes compared to non-SAO children, high level (>90% binding inhibition) *P. vivax* Duffy binding protein–specific binding inhibitory antibodies were observed significantly more often in sera from SAO than non-SAO children (SAO, 22.2%; non-SAO, 6.7%; *p* = 0.008).

**Conclusions:**

In three independent studies, we observed strong associations between SAO and protection against *P. vivax* malaria by a mechanism that is independent of the Duffy antigen. *P. vivax* malaria may have contributed to shaping the unique host genetic adaptations to malaria in Asian and Oceanic populations.

*Please see later in the article for the Editors' Summary.*

## Abstract 2

Ivo Mueller and colleagues examined the association of Southeast Asian ovalocytosis with *Plasmodium vivax* infection by genotyping 1975 children enrolled in three independent epidemiological studies conducted in the Madang area of Papua New Guinea and assessing *P. vivax* infection and disease in the children.

## Editors' Summary

**Background:**

Hereditary blood disorders are widely prevalent in different regions around the world and the type of disorder depends on the population gene pool. For example, sickle cell disease is indigenous to sub-Saharan Africa, and Southeast Asian ovalocytosis (SAO), as the name suggests, to Southeast Asia and the South West Pacific, particularly Malaysia and Papua New Guinea. In SAO, the red blood cells (erythrocytes) are a different shape (elliptical) from the usual biconcave disc shape due to a genetic defect (caused by band 3 deletion *SLC4A1Δ27*) in the red blood cell membrane. This defect is carried by up to 35% of people living on the coasts of Papua New Guinea, and as these areas match high malaria endemic areas, it is thought that carrying this defect is associated with improved survival against malaria in these populations—some studies have suggested that SAO is associated with complete protection against cerebral malaria but not other forms of malaria caused by the same type of parasite—*P. falciparum*.

**Why Was This Study Done?:**

Although *P. falciparum* gains most attention by the international health community as it causes the most severe types of malaria, recent epidemiological studies suggest that malaria caused by *P. vivax* can also cause severe illness in some areas of the world where it is highly prevalent. Furthermore, detailed genetic and laboratory studies have suggested that the genetic defect associated with SAO may actually protect against infection from *P. vivax* malaria. So in this study, the researchers examined the relationship of the SAO genetic defect and *P. vivax* malaria by doing genetic tests on children in Papua New Guinea—an area in which both conditions are widely prevalent.

**What Did the Researchers Do and Find?:**

The researchers performed genetic tests for the SAO defect in 1,975 children in the Madang area of Papua New Guinea who were participating in three separate malaria studies that were conducted over different time periods: (i) a cohort of 1,121 infants aged 3–21 months participating in a clinical drug trial of intermittent preventative treatment of malaria; (ii) a case-control study of 318 children with severe malaria aged 10 years and under; and (iii) a cohort of 206 children aged 5–14 years who took part in a prospective study to evaluate the time of reinfection with all forms of malaria. Given the different nature of these studies, for example, the effect of intermittent treatment for which the researchers had to make statistical adjustments, the researchers analysed the presence of the SAO genetic defect and the incidence of all forms of malaria separately to calculate the association with SAO and malaria in the participants in each study.

The researchers found that the SAO genetic defect present in 130 infants (11.6%) in the first study and 27 (13.1%) children in the third study. In the case-control study, the researchers found that 28 of the 330 controls (8.5%) had the SAO genetic defect compared to eight of 236 (3.4%) in children with *P. falciparum* single infections. Overall, the researchers found that the SAO genetic defect was associated with a 43% reduction in risk of clinical *P. vivax* episodes in the infants in the first study and a 52%–55% reduction in *P. vivax* reinfection in children in the third study. Furthermore, from the limited data from the second study, the researchers found that none of the children with *P. vivax* or mixed *P. falciparum*/*P. vivax* malaria had the SAO defect, which may indicate a protective effect.

**What Do These Findings Mean?:**

These findings suggest that the SAO genetic defect (*SLC4A1Δ27*) may have a protective effect against malaria caused by *P. vivax* in infants and children of different ages in Papua New Guinea. However, although it seems likely that SAO may alter the ability of the malarial parasite to develop within the red blood cell, this study sheds no further light on the way in which the SAO genetic defect may protect against *P. vivax* infection and disease and so further studies are needed to investigate possible mechanisms. Importantly, these findings suggest that future studies investigating genetic adaptation of diverse populations around the world, particularly in the Asian Pacific region, should include all forms of human malaria, such as *P. vivax*, and not exclusively focus on *P. falciparum*.

**Additional Information:**

Please access these Web sites via the online version of this summary at http://dx.doi.org/10.1371/journal.pmed.1001305.

- Wikipedia has information on Southeast Asian Ovalocytosis (note that Wikipedia is a free online encyclopedia that anyone can edit; available in several languages)
- WHO provides information on malaria
- The US Centers for Disease Control and Prevention provide information on malaria (in English and Spanish)
- MedlinePlus provides links to additional information on malaria and on ovalocytosis

## Introduction

The populations of the southwest Pacific are highly diverse and exhibit a range of unique red blood cell (RBC) polymorphisms. Within Papua New Guinea (PNG), a variety of RBC variants are found that have geographical patterns paralleling malaria endemicity [1], suggesting selective pressure by this disease [2]. In particular, Southeast Asian ovalocytosis (SAO) (caused by band 3 deletion *SLC4A1Δ27*) has a distribution that is closely correlated with malaria prevalence [3]. Even though the *SLC4A1Δ27* deletion is lethal in the homozygous state [4], prevalence of heterozygosity reaches 35% in some PNG coastal populations [3]. Therefore, the *SLC4A1Δ27* deletion is thought to be associated with improved survival against malaria in these populations. Indeed, SAO has been associated with complete protection against cerebral but not other forms of severe *P. falciparum* malaria in previous epidemiological studies in PNG [5]. SAO was, however, found to have little or no association with reduced prevalence, parasitaemia, or uncomplicated *falciparum* malaria [6]–[8].

Although non-*falciparum* parasites are often considered to cause only mild disease, recent data from the island of New Guinea [9]–[11], Brazil [12], and India [13] show that *P. vivax* infections are associated with severe disease and mortality. In addition, mortality rates of 5% to 15% were regularly observed in patients challenged with *P. vivax* for therapy of tertiary syphilis [14]–[18]. Thus, *P. vivax* may be responsible for, or contribute to, natural selection of erythrocyte polymorphisms. This selective pressure is suggested by the emergence of the unique, non-African Duffy-negative allele (FY*A^ES^) in PNG and the observation that PNG children expressing heterozygotes (FY*A/FY*A^ES^) are protected from *P. vivax* blood-stage infection [19]. The proposed Austronesian origin of *SLC4A1Δ27* [20], and its geographical restriction to Southeast Asia and the southwest Pacific, regions that are co-endemic for all four human malaria parasite species, led us to hypothesize that *P. vivax* malaria could have contributed to selection of this genetic polymorphism. An early cross-sectional [21] and a case-control study [6] indicated that Melanesians with hereditary ovalocytosis experienced lower parasitaemia and frequency of infection with *P. vivax* and/or *P. malariae*. While three field studies have investigated associations between *SLC4A1Δ27* and prevalence of *P. vivax* blood-stage infection, no significant relationship with susceptibility to infection was observed [7],[22],[23]. However, these were small cross-sectional studies with insufficient statistical power to unmask an association.

In vitro studies have shown that SAO red cells are relatively resistant to invasion by some *P. falciparum* isolates [24],[25], with the degree of resistance influenced by the “receptor preferences” of the isolate [26] and deformability of the red cell membrane [25]. Ovalocytes exhibit reduced susceptibility to invasion by *P. knowlesi* in vitro, with the suggestion that this is mediated by diminished adherence [27]. These observations indicate that SAO may protect against infection with *P. vivax* malaria in vivo.

In order to assess the relationship between SAO (i.e., *SLC4A1Δ27*) and risk of infection and/or disease with *P. vivax*, we genotyped 1,975 children participating in three separate studies conducted in the Madang area of PNG: (i) a cohort of infants participating in a clinical trial of intermittent preventative treatment (IPTi) [28], (ii) a pediatric severe malaria case-control study [29], and (iii) a cohort of children aged 5–14 y that took part in a prospective longitudinal cohort study in which all individuals were initially treated to clear blood-stage infection and subsequently evaluated for a delay in time-to-reinfection by all *Plasmodium* species [30].

## Methods

### Description of Field Studies

#### Infant cohort

Between July 2006 and June 2009, a total of 1,121 infants 3 mo of age were enrolled in a randomized, placebo-control trial of intermittent preventive treatment for the prevention of malaria and anemia [28]. After screening, consent, and enrolment children were randomized to receive a full treatment course of either amodiaquine+sulphadoxine/pyramethamine (SP), artesunate+SP, or placebo at 3, 6, 9, and 12 mo. A total of 1,079 children completed follow-up until 15 mo of age, with an additional 857 followed to 21 mo. At each treatment/follow-up time point, bednet usage was assessed, recent antimalarial treatment documented, and a 250-µl finger-prick blood sample was collected from all children for later detection of infection (by light microscopy [LM] and post-PCR ligase detection reaction-fluorescent microsphere assay [LDR-FMA] [31]). A full clinical examination was only conducted on children spontaneously reporting signs of clinical illness. A passive case detection system was maintained at the local health centre, three associated aid posts, and a system of monthly study clinics for the entire study period.

All children presenting with self-reported signs of a febrile illness were promptly assessed for presence of malaria infection by rapid diagnostic test (RDT) (ICT Combo test), and only RDT positive children treated with arthemeter-lumefantrine (Coartem, Novartis). A finger-prick sample was collected from all febrile cases for confirmation of malarial infections by LM and PCR. For clinical management and analysis purposes, malarial illness was defined as axillary temperature >37.5°C, or history of fever in preceding 48 h, plus an infection of any density by LM and/or a positive RDT with speciation subsequently confirmed by PCR. A more detailed description of the study and its primary outcomes is given elsewhere [28].

#### Pediatric severe malaria case-control study

Between October 2006 and December 2009, a total of 318 children of Madang or Sepik parentage aged 0.5–10 y admitted to the pediatric ward of Modilon Hospital, Madang Town with a diagnosis of severe malaria and 330 age-, location-, and ethnicity- matched healthy controls were enrolled in a case-control study designed to investigate the associations of host genetic polymorphism with protection against severe malaria. Cases were defined as severe malaria if they fulfilled the World Health Organization (WHO) definition of severe malarial illness [32]. Inclusion criteria included any of: (i) impaired consciousness/coma (Blantyre Coma Score [BCS]<5 [23]); (ii) prostration (inability to sit/stand unaided); (iii) multiple seizures; (iv) hyperlactatemia (blood lactate >5 mmol/l); (v) severe anemia (hemoglobin <50 g/l); (vi) dark urine; (vii) hypoglycemia (blood glucose <2.2 mmol/l); (viii) jaundice; (ix) respiratory distress; (x) persistent vomiting; (xi) abnormal bleeding; or (xii) signs of shock [29]. Where clinically indicated, CSF (*n* = 124) and blood (*n* = 281 cultures were taken on admission. All bacterial cultures from severe malaria cases were sterile [29]. Healthy community controls were recruited from the village of origin of the matched cases. At enrolment, a blood sample for determination of malaria (by LM and nested PCR [33]) and for host genotyping was collected from each case and control. A detailed description of all study procedures and in-depth clinical description of all cases is given elsewhere [29]. As specified in the study protocol, the primary case definition of a severe malaria case for analysis of host genetic associations included the following additional criteria: parasitaemia (>1,000 *P. falciparum*/µl or >500 *P. vivax*/µl) and parents from the PNG North Coast (Madang, Morobe, and Sepik) (http://www.malariagen.net/node/242). Consequently, severe cases with mixed *P. falciparum*/*P. vivax* infections by LM or PCR were only included as mixed cases if both species exceeded their respective thresholds. The parasitaemia cut-offs were based on local attributable fraction-based definitions of malaria episodes [34] and were included to increase the specificity of case definition.

#### Treatment time-to-reinfection study

The study population of 206 children for this treatment time-to-reinfection study has been described previously [30]. Briefly, 206 elementary and primary school students from Madang Province (Mugil and Megiar) participated in this study conducted from June to December, 2004. At enrolment, a peripheral venous blood sample was collected for determination of *Plasmodium* species infection status by LM and LDR-FMA. All children irrespective of infection status were treated with a 7-d course of artesunate monotherapy (4 mg/kg on day 1 and 2 mg/kg on days 2–7) that successfully cleared all but one *P. vivax* infection [30]. As no primaquine was given, subsequent *P. vivax* reinfections of the blood stream can either be from newly acquired infection via sporozoites or relapsing infections from hypnozoites.

Following treatment, children were followed up by active surveillance every 2 wk and passive surveillance at the local health centre for a total of 25 wk. Children were monitored for acquisition of new infections until they completed follow-up, withdrew from the study, or did not provide two consecutive bi-weekly blood samples. At the time of the study, bednet usage in the area was limited, most nets were untreated, and their use was not associated with differences in risk of reinfection [30].

At each bi-weekly follow-up visit, a 250-µl finger-prick blood sample was collected from each child for detection of malaria by LM and LDR-FMA. If a child presented with clinical malaria symptoms at a follow-up visit or during the intervening period, a peripheral venous blood sample was taken and treatment given in accordance with 2000 PNG guidelines (amodiaquine [3 d] plus sulfadoxine/pyrimethamine on day 1).

These studies were reviewed and approved by institutional review boards of PNG Medical Research Advisory Council, the IMR Institutional Review Board, the Walter & Eliza Hall Institute, and the Veterans Affairs Medical Center (Cleveland, Ohio, US).

### Detection of *Plasmodium* Species Infection

Standard procedures were used for reading the blood smears and estimating parasite densities [28]–[30]. All blood smears were read independently by two experienced microscopists with parasites counted against 200 white cells. Discrepant results were adjudicated by a third microscopist. Blood smears were scored as LM positive for an individual *Plasmodium* species if the species was detected independently by at least two microscopists and/or subsequent PCR-based analysis confirmed the presence of the species. Densities were converted to the number of parasites per µl of blood assuming 8,000 white blood cells/µl [35].

DNA was extracted using the QIAamp96 DNA Blood kit (Qiagen) from all blood samples. Infection by each of the four human malaria species co-endemic in PNG (*P. falciparum*, *P. vivax*, *P. malariae*, and *P. ovale*) was assessed in all blood samples collected in the two cohorts using a semi-quantitative post-PCR LDR-FMA [31] and by nested PCR (nPCR) [36] in samples from the case-control study. Both assays are based on the amplification of the small subunit (SSU) ribosomal RNA gene.

### PCR-Based Genotyping

Deletion and SNP genotyping of *SLC4A1Δ27* (Chromosome 17) associated with SAO, glycophorin C exon 3 deletion (*GYPCΔex3*; Chromosome 2) associated with Gerbich-negativity [37],[38], the common Melanesian 3.7 kb (α^−3.7^) and 4.2 kb (α^−4.2^) α-globin (Chromosome 16) deletions associated with α^+^-thalassaemia [39], and Duffy blood group polymorphisms were all performed using methods that have been described previously [40].

### Flow Cytometry-Based Analyses of Duffy Antigen on RBCs

Twelve of 21 SAO children participating in the treatment time-to-reinfection study agreed to provide an additional finger-prick blood sample for flow cytometry-based analyses of Duffy antigen on their red cells. For each SAO child, a control sample was collected from a non-SAO (wild-type) child living in the same hamlet. All samples collected were separated into plasma and cell pellets. One sample from an SAO child was subsequently discarded because of excessive cell lysis. Pelleted blood was suspended in 1 ml of 10% DMSO+90% fetal calf serum solution and frozen to −80°C. Prior to antibody staining and protein binding, cells were rapidly thawed for 1 min in 37°C water bath and washed (2×) by first resuspending the red cell pellet in 1.0 ml of RPMI-1640 followed by centrifugation at 600*g* for 10 min. In order to assess Duffy receptor RBC surface expression for individual blood donors, 1×10^6^ RBC from each sample were incubated with 50 µl (1∶50) of mouse anti-human Fy6 antibody for 15 min at 37°C. Cells were then washed with RPMI-1640, stained with PE-conjugated goat anti-mouse antibody (Molecular Probes), and analyzed using a Becton Dickinson LSR II (Franklin Lakes).

To assess possibility that there are differences in the ability of *P. vivax* parasites to bind to SAO versus non-SAO RBC, we evaluated the level of *P. vivax* Duffy binding protein (PvDBP) binding using the same set of blood samples as described above [41]. Briefly, 1×10^6^ washed RBC from each sample were re-suspended in 200 µl of RPMI-1640 and exposed to 0.5 µg of recombinant PvDBP region II (PvDBPII, Sal I variant) for 15 min at 37°C. Excess protein was removed by centrifugation of erythrocytes at 3,000*g* for 2 min, resuspended in RPMI-1640 followed by centrifugation at 3,000*g* for 2 min (2×), and then resuspended in 100 µl of RPMI-1640 to which rabbit anti-PvDBPII antibody (1∶5,000 final dilution) was added and incubated for 30 min at 37°C, washed (2×) as above and resuspended in 100 µl of RPMI-1640 to which 1∶4 to 1∶16 dilution of PE-conjugated goat anti-rabbit antibody (Sigma-Aldrich Co., depending on the lot of conjugated antibody). All data were evaluated as normalized mean fluorescence index (nMFI = % of positives cells×MFI) [42].

### Measurement of Antibody Titers to Recombinant PvDBPII Variants and Blocking Antibodies

Measurement of binding inhibitory antibodies (BIAbs) and total antibodies to PvDBPII PvMSP1, PvRBP1, PvRBP2, and PvRBP3 was performed as previously described [43],[44].

### Statistical Analyses

The association of malaria incidence rates with SAO genotype in children 3–21 mo was assessed using negative binomial regression. Analyses were adjusted for gender, treatment effect, season (wet versus dry), and village of enrolment (grouped by 12 recruitment zones). The time-at-risk was calculated starting on the date of enrolment until the child either reached the final study time point at 21 mo of age, or was withdrawn from the study [45]. Associations between SAO and the prevalence and density of *P. vivax* and *P. falciparum* infections were investigated using generalized estimating equation models (XTLOGIT and XTREG in STATA 10.0) that allowed accurate assessment of both variations in the outcome and the correlations between repeated measurements in individual children (modeled using an exchangeable correlation structure). A semi-robust Huber/White/sandwich estimator of variance was used to assure valid standard errors. Best fitting models were determined by backward elimination using Wald's Chi-square tests for individual variables.

In the study of children 5–14 y, log-rank tests were used to assess differences in Kaplan-Meier curves of time-to-first reinfection as detected by LDR-FMA or LM. Cox regression was employed to test for differences in time-to-first reinfection after adjusting for all other factors that were found to be associated with difference in time-to-first reinfection [30]. In these analyses children were considered at risk of acquiring a *Plasmodium* spp. infection until they either reached the end point, missed two consecutive bi-weekly follow-ups, were re-treated with antimalarials, or withdrew from the study.

Difference in frequency of SAO genotype among severe malaria cases and controls were tested using χ^2^ tests. Association of SAO genotype with other common RBC polymorphisms and potential confounders were assessed using χ^2^ and Student *t*-tests (for continuous, normally distributed variables). The associations between different genetic traits and prevalence of BIAbs against PvDBPII were assessed using Chi-square and Fisher exact tests. Due to non-normality, differences in both expression of the Duffy receptor on RBC and in binding of recombinant PvDBPII on SAO and non-SAO RBCs were compared using non-parametric Mann-Whitney U-tests.

All 95% confidence intervals are model based. Further details on statistical approaches employed are given elsewhere [30],[45]. All statistical analyses were performed using STATA 8 & 10 (Stata Corporation) statistical analysis software.

## Results

### Association of SAO with Incidence of Clinical Malaria

A total of 1,121 children (48.3% female) were enrolled in the IPTi trial, with equal numbers in each treatment arm. Of these, 857 were followed up to 21 mo. Over the entire 18-mo follow-up period, children on average experienced 0.74 *P. vivax* and 0.28 *P. falciparum* episodes/child/year. IPTi with amodiaquine-SP reduced the incidence of *P. vivax* by 23% (95% CI 0–41, *p* = 0.048) for the first year of follow-up (3–15 mo) and incidence of *P. falciparum* by 35% (95% CI 9–54, *p* = 0.012), whereas IPTi with artesunate-SP only protected against *P. falciparum* (31%, 95% CI 4–51, *p* = 0.027) but not against *P. vivax* episodes (6%, 95% CI −24 to 26, *p* = 0.759) [28]. No significant differences were observed between all three arms for the 15–21-mo extended follow-up period (*p*>0.75).

SAO genotypes were available for all 1,121 children with 130 heterozygous for *SLC4A1Δ27* (11.6%), with no significant difference in frequency of SAO among treatment arms (*p* = 0.30). *SLC4A1Δ27* heterozygocity was associated with neither α+thalassaemia nor Gerbich-negative deletion (*GPYCΔex3*, *p*>0.95) (Table S1). There were no differences between SAO and wild-type children in bednet usage or village of residence, gender, or season of recruitment (*p*>0.36).

Over the total follow-up period (3–21 mo), SAO was associated with a significant 32% reduction in incidence of any form of malaria (95% CI 11–49, *p* = 0.0068) (Table 1). The protection was exclusively directed against episodes of *P. vivax* malaria, with the SAO genotype associated with 43% reduction (95% CI 22–59, *p* = 0.0006) in incidence of *P. vivax* episodes of any density and a 55% reduction (95% CI 34–59, *p*<0.0001) in incidence of *P. vivax* episodes with >500 parasites/µl. There were no significant associations with all *P. falciparum* episodes (incidence rate ratio [IRR] = 1.03, 95% CI 0.73–1.45, *p* = 0.89) (Table 1) or episodes with *P. falciparum* >2,500 parasites/µl (IRR = 1.03, 95% CI 0.69–1.55, *p* = 0.86).

**Table 1:** Associations between SAO and incidence of malaria during follow-up in infants 3–21 mo.

Clinical Malaria	Wild Type (*n* = 991)			SAO (*wt/Δ27*, *n* = 130)			IRR^a^	95% CI	*p*-Value
	Events	PYAR	Incidence	Events	PYAR	Incidence			
All episodes	1,309	1,288.6	1.02	122	173.0	0.71	0.68	0.51–0.89	0.0062
All Pf episodes	373	1,359.0	0.27	51	178.3	0.29	1.03	0.73–1.46	0.86
Pf>2,500/µl	242	1,368.7	0.18	33	179.7	0.18	1.04	0.69–1.55	0.86
All Pv episodes	1,018	1,309.8	0.78	80	176.1	0.45	0.57	0.41–0.78	0.0005
Pv>500/µl	800	1,326.2	0.60	50	178.3	0.28	0.45	0.31–0.66	<0.0001

a IRR-AHRs with analyses adjusted for the following potential confounders: gender, village of residence, average bednet usage, season of recruitment, and IPTi treatment group.

PYAR, person year at risk.

### SAO and Prevalence and Density of Malaria Infection

**Figure 1:**
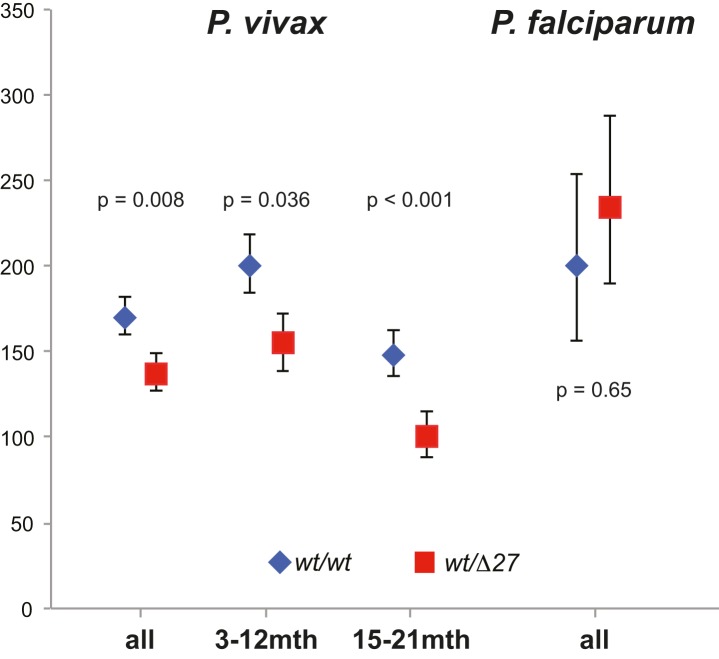
Parasite density (by LM) in species in SAO (wt/Δ27, red squares) and non-SAO children (wt/wt, blue rhombi). Significance levels adjusted for IPTi treatment, insecticide-treated net use, and village of residence.

In children 3–21 mo, the prevalence and density of *P. vivax* and *P. falciparum* were evaluated in 6,269 blood samples collected at scheduled 3-monthly study contacts. Of these, 1,435 (22.9%) and 352 (5.6%) were positive for *P. vivax* and *P. falciparum*, respectively, by LDR-FMA and 843 (12.9%) and 197 (3.0%) by LM. Infections with *P. malariae* and *P. ovale* were rare even by LDR-FMA (1.3% and 0.5%, respectively). There was no significant difference in the prevalence *P. vivax* infections in SAO and wild-type children ≤12 mo (Table 2). However, in *SLC4A1Δ27* heterozygous children aged 15–21 mo, significantly fewer *P. vivax* infections were detected by both LDR-FMA (adjusted odds ratio [aOR] 0.71, *p* = 0.041) and LM (aOR 0.39, *p* = 0.001). In children of all ages, *P. vivax* parasite densities (by LM) were significant lower in *SLC4A1Δ27* heterozygous children (1,111/µl versus 636/µl, *p* = 0.011) (Figure 1). By contrast, the prevalence of *P. falciparum* was higher in *SLC4A1Δ27* heterozygous children (reaching statistical significance only for LDR-FMA positive infections) (Table 1), but parasite densities were comparable (1,642/µl versus 2,104/µl, *p* = 0.59).

**Table 2:** Associations between SAO and prevalence of *P. vivax* and *P. falciparum* infection in infants 3–21 mo.

Species and Age Groups	Genotype	LDR-FMA^a^			LM^b^		
		PR	aOR^c^	95% CI	PR	aOR^c^	95% CI
***P. vivax***							
**All children**	wt/wt	23.1%	—	—	13.1%	—	—
	*wt/Δ27*	19.7%	0.84	0.66–1.05	9.7%	0.72	0.50–1.04
		—	—	(*p* = 0.13)	—	—	(*p* = 0.08)
**3–12 mo**	wt/wt	19.1%	—	—	9.6%	—	—
	*wt/Δ27*	17.8%	0.93	0.69–1.25	10.4%	1.12	0.75–1.68
		—	—	(*p* = 0.64)	—	—	(*p* = 0.59)
***15–21 mo***	wt/wt	30.3%	—	—	18.9%	—	—
	*wt/Δ27*	23.1%	0.72	0.51–0.99	8.5%	0.39	0.23–0.67
		—	—	(*p* = 0.044)	—	—	(*p* = 0.001)
***P. falciparum***							
**All children**	wt/wt	5.2%	—	—	2.9%	—	—
	*wt/Δ27*	8.4%	1.65	1.17–2.32	4.1%	1.51	0.97–2.36
		—	—	(*p* = 0.004)	—	—	(*p* = 0.067)
**3–12 mo**	wt/wt	4.0%	—	—	2.4%	—	—
	*wt/Δ27*	6.7%	1.67	1.07–2.62	3.5%	1.45	0.85–2.48
		—	—	(*p* = 0.024)	—	—	(*p* = 0.18)
***15–21 mo***	wt/wt	7.3%	—	—	3.6%	—	—
	*wt/Δ27*	11.4%	1.62	1.00–2.64	5.1%	1.41	0.71–2.81
		—	—	(*p* = 0.052)	—	—	(*p* = 0.32)

a Infections diagnosed by post-PCR LDR-FMA.

b Infections diagnosed by expert LM.

c AORs with analyses adjusted for the following variables: IPTi treatment group, insecticide treatment bednet usage, village of residence.

PR, prevalence rate.

Of the 206 children 5–14 y of age enrolled in the treatment-reinfection cohort 106 (51.5%) were female; 116 (56.9%) were over 8 y of age. 27 children (13.1%) were heterozygous for *SLC4A1Δ27*. *SLC4A1Δ27* heterozygous children, were similar in the basic characteristics to wild-type children except that significantly more of them were attending Mugil Elementary School (96.3% versus 70.4%, *p* = 0.004) (Table S2). There were no significant associations between SAO and α^+^-thalassaemia or *GPYCΔex3* genotypes [46]. Genotype frequencies did not differ with age and sex (Fisher exact test, *p*>0.4 [8]). All children were wild-type (FY*A/FY*A) for the Duffy antigen.

Following initial blood-stage malaria therapy, children rapidly acquired LM-detectable blood-stage infections, with 156/192 (81.2%), 102/206 (49.5%), and 17/206 (8.3%) acquiring one or more *P. falciparum*, *P. vivax*, and *P. malariae* infections, respectively, over the 26 wk of observation. Similarly, using LDR-FMA diagnosis, the proportions of reinfected children rose to 91.6%, 82%, and 29.3%, respectively [30].

**Figure 2:**
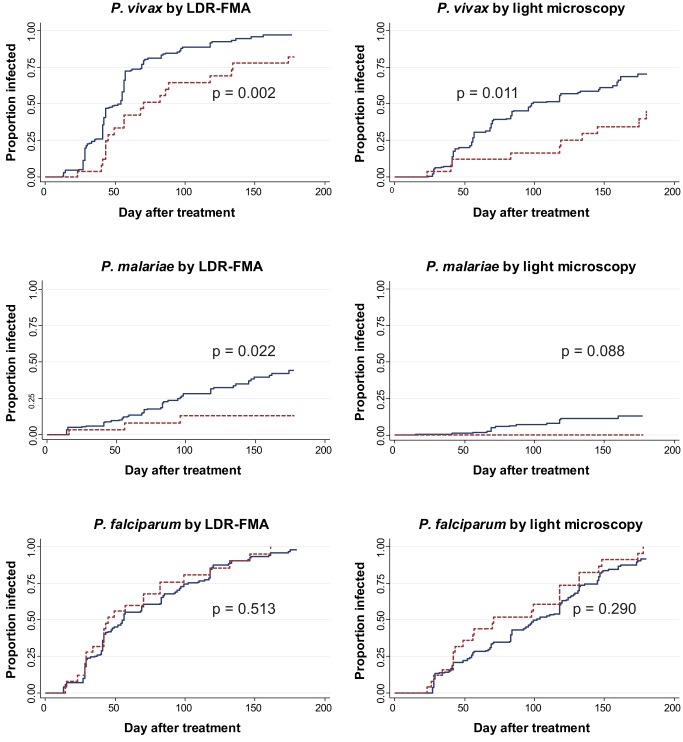
Time-to-first blood-stage infections with different Plasmodium species in SAO (dashed) and non-SAO children (solid). Kaplan-Meier Curves with log-rank test for difference.

While *SLC4A1Δ27* heterozygous and wild-type children had similar times to reinfection with *P. falciparum* [8], a significantly lower proportion of heterozygous children acquired *P. vivax* and *P. malariae* reinfections during the follow-up time period (Figure 2). Among *SLC4A1Δ27* heterozygotes, 70.3% (19/27) acquired at least one LDR-FMA-positive *P. vivax* infection compared to 83.8% (149/179) of non-SAO children (log rank test, χ^2^ = 9.68, df = 1, *p* = 0.002) over the period of observation. Similarly, *P. vivax* infections were observed in only 37% (10/27) of *SLC4A1Δ27* heterozygotes by microscopy, compared to 54.7% (98/179) of non-SAO children (log rank test, χ^2^ = 6.5, df = 1, *p* = 0.011). After correction for all other factors found to be associated with differences in risk of reinfection [30], *SLC4A1Δ27* heterozygosity was associated with a significant 52% reduction in time to first LDR-FMA (adjusted hazard ratio (aHR): 0.48, *p* = 0.003) (Table 3) and 55% reduction in time to first LM-detectable *P. vivax* infections (aHR: 0.45, *p* = 0.014), as determined by Cox regression analysis. Similarly, SAO was associated with a 71% reduction in time to first LDR-FMA-detectable (aHR: 0.29, *p* = 0.03) *P. malariae* infections. SAO was however not associated with protection against *P. falciparum* (Table 3) [8].

**Table 3:** Associations between SAO and to first *Plasmodium spp.* infection during follow-up in children 5–14 y.

Species	Genotype	*n*	LDR-FMA^a^ aHR^c^	95% CI	LM^b^ aHR^d^	95% CI
***P. vivax***	wt/wt	179	—	—	—	—
	*wt/Δ27*	27	0.48	0.29–0.78	0.45	0.23–0.87
		—	—	(*p* = 0.003)	—	(*p* = 0.014)
***P. malariae***	wt/wt	179	—	—	—	—
	*wt/Δ27*	27	0.29	0.09–0.91	0.0^e^	—
		—	—	(*p* = 0.033)	—	(*p* = 0.0)^e^
***P. falciparum***	wt/wt	168	—	—	—	—
	*wt/Δ27*	25	1.15	0.75–1.76	1.32	0.85–2.04

a Infections diagnosed by post-PCR LDR-FMA.

b Infections diagnosed by expert LM.

c AHRs with analyses adjusted for the following significant confounders: *P. vivax*, presence of LM+ Pv infection at baseline; *P. falciparum*, distance from residence to local health centre; *P. malariae*, none.

d AHRs with analyses adjusted for the following significant confounders: *P. vivax*, age >9 y; *P. falciparum*, distance from residence to local school elementary school+LDR-FMA positive Pf infection at baseline; *P. malariae*, distance from residence to local health centre and to local elementary school.

e As LM-positive *P. malariae* infection were observed among SAO children, confidence interval could not be estimated and *p*-value obtained by log-rank test.

### Duffy Expression and PvDBP Binding on SAO Red Cells

**Figure 3:**
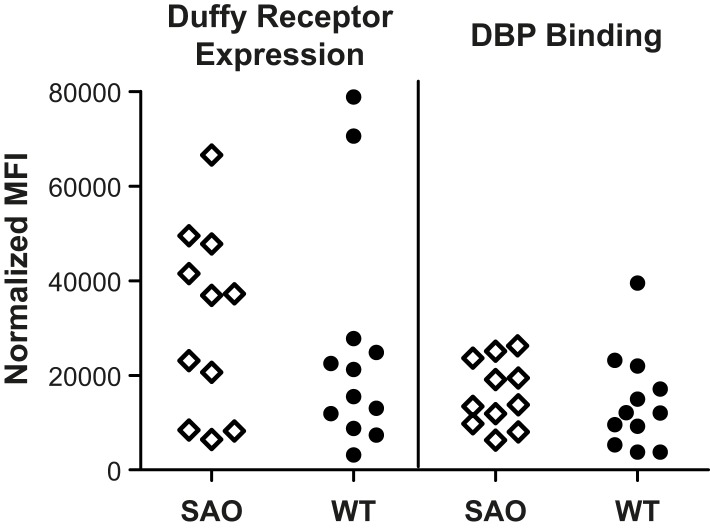
Duffy receptor expression on erythrocytes as measured by mAb Fy6 on SAO and non-SAO cells (left panel) and binding of PvDBPII to its' receptor on SAO and non-SAO cells (right panel). Each spot represents analysis of 5×10^5^ erythrocytes from a single donor.

A possible mechanism whereby *SLC4A1Δ27* may confer protection against *P. vivax* may be through reduced expression and altered functionality of the Duffy antigen receptor on RBCs. Accordingly, levels of Duffy expression and ability to bind recombinant PvDBPII were compared on red cells from a subset SAO (*n* = 11) and non-SAO (*n* = 12) study children. Erythrocytes with *SLC4A1Δ27* showed no significant decrease in expression of the Duffy receptor on the surface (Figure 3, left panel, mean ± standard error of the mean [SEM] normalized mean fluorescence index [nMFI] = 36,881, inter-quartile range [IQR] 8,455–47,750, versus 18,360, IQR 9,555–27,040, *p* = 0.42 for SAO and non-SAO cells, respectively). The ability of PvDBPII to bind to the different SAO and non-SAO cells was similar (nMFI = 13,755, IQR 9,757–23,603, versus 12,069, IQR 6,292–20,740, *p* = 0.36) (Figure 2, right panel).

### SAO and Generation of *P. vivax*-Specific Antibody Responses

Since we had previously shown that high binding inhibitory antibodies to PvDBPII (>90% inhibitory activity) correlated with protection against *P. vivax* in this population [43], we examined whether SAO affects a generation of protective anti-DBPII antibodies. Among *SLC4A1Δ27* heterozygous children 6/27 (22.2%) had high levels of PvDBPII BIAbs (>90% strain-transcending binding inhibition [43]) compared to 12/179 (6.7%, *p* = 0.008) in non-SAO children. Adjusting for the presence of PvDBP-specific BIAbs did not significantly alter the strength of protection of SAO against *P. vivax* infections detected by either LDR-FMA (aHR = 0.49, 95% CI 0.30–0.81, *p* = 0.008) or LM (aHR = 0.49, 95% CI 0.25–0.95, *p* = 0.036).

In contrast to the above findings, total IgG antibodies as measured by ELISA to the main variants of PvDBPII (AH, O, and P) as well as to other *P. vivax* merozoite surface proteins (PvRBP1, PvRBP2, and PvMSP1_19_) were not significantly different in SAO compared with non-SAO children (unpublished data).

### Prevalence of SAO among Severe Malaria Cases

Of 318 cases fulfilling the standard WHO definition of severe malaria, 273 were infected with *P. falciparum*, 34 with *P. vivax*, ten with *P. falciparum* plus *P. vivax*, and one with *P. malariae*. Of these 264 (83.0%) also fulfilled the pre-defined, more stringent criteria (parasitaemia cut-offs plus of local Madang or Sepik parentage) for the host genetic study: 236 were infected with *P. falciparum* (>1,000/µl), 25 with *P. vivax* (>500/µl), two with *P. falciparum* plus *P. vivax*, and one with *P. falciparum* plus *P. malariae*. *SLC4A1Δ27* genotyping was successfully preformed in cases and healthy community controls.

28 of 330 (8.5%) health community controls were heterozygous for *SLC4A1Δ27* compared with eight of 236 (3.4%) in children with *P. falciparum* single infections (OR = 0.38, 95% CI 0.15–0.87, *p* = 0.014) and 0/27 (0%) for children with *P. vivax* or mixed *P. vivax/P. falciparum* infection (OR = 0, 95% CI 0–1.56, *p* = 0.11, exact test).

When all cases fulfilling WHO criteria for severe malaria are considered, two cases of *P. vivax* infection (case 1: 429 parasites/µl with prostration and mildly impaired consciousness (Blantyre coma score = 4); case 2: 98 parasites/µl (mixed with *P. falciparum* by PCR), severe anemia (Hb = 1.7 g/dl), and hyperlactemia (lactate = 6.3 mmol/l) were observed in children with SAO genotype.

Among the eight SAO children with *P. falciparum* mono-infection, three presented with deep coma (Blantyre coma scores of 2, 1, and 2 respectively) in presence of *P. falciparum* densities of 4,266, 219, and 309/µl. All three children had clear CSF and negative CSF and blood cultures. These three cases are consistent with the accepted clinical definitions for cerebral malaria caused by *P. falciparum* [23],[29]. A detailed clinical description of each case is given in Text S1.

## Discussion

This study shows that a deletion in an RBC integral membrane protein *SLC4A1Δ27* causing SAO was associated with a 43% reduction in risk of clinical *P. vivax* episodes in a large cohort of infants followed from age 3 to 21 mo and a 52%–55% reduction in *P. vivax* reinfection diagnosed by PCR-LDR-FMA and LM, respectively, in a cohort of children aged 5–14 y. This is the first in vivo evidence that SAO may afford partial protection against *P. vivax* malaria in two independent, longitudinal cohort studies. In addition, SAO was associated with a lower *P. vivax* parasitaemia in children aged 3–21 mo and a reduced prevalence of *P. vivax* infections in children 15–21 mo. Last but not least, no child SAO genotype was found among 27 cases with severe *P. vivax* or mixed *P. falciparum*/*P. vivax* malaria. However, SAO was not associated with protection against *P. falciparum* infection and uncomplicated disease. While it was associated with a decreased risk of severe *P. falciparum* malaria, at least one severe malaria case with SAO genotype was admitted with deep coma (Blantyre Coma score = 2), refuting the earlier assertion that SAO provides complete protection against cerebral *P. falciparum* malaria [5],[47].

SAO is a dominant phenotype with respect to RBC morphology. All individuals who are *SLC4A1Δ27* heterozygous have erythrocytes that are ovalocytic and more rigid than normal [48]. Although it has been suggested that the mutated band 3 protein retains its normal secondary structure [49], the membrane domain is modified [49] and the variant protein does not conduct anion transport normally [50]. While SAO erythrocytes exhibit approximately half of the anion transport activity of normal erythrocytes [50], this does not contribute to anemia or significantly impair erythrocyte function [51]. Despite being lethal in its homozygous state [4], the prevalence of heterozygosity reaches 35% in some coastal populations in PNG [3], indicating a strong selective advantage of the heterozygous genotype. Until now protection against *P. falciparum* cerebral malaria has been considered the most likely cause of selection for SAO [5],[47]. The strong reduction in the incidence of *P. vivax* disease and infections observed in our study opens up the possibility that protection against *P. vivax* malaria could at least have contributed to the heterozygote advantage of SAO in PNG.

It is now well established that both *P. falciparum* and *P. vivax* are associated with severe disease and death in Melanesian populations [9],[10],[29],[52]. In our studies in PNG, although *P. vivax* infections were less likely to result in severe symptoms, children admitted with severe *P. vivax* had the same phenotype as those with severe *P. falciparum* infections, while those with mixed *P. falciparum*/*P. vivax* presented with the most severe illness and the greatest mortality [29]. Absence of any cases of severe vivax malaria with a parasitaemia >500/µl indicates that SAO could result in a *P. vivax*-specific mortality benefit. However, the limited number of both SAO children and severe *P. vivax* infections in our case-control study restricts our ability to assess this association. Larger, appropriately powered studies are therefore required to confirm this finding.

The mechanism by which SAO may protect against *P. vivax* infection and disease is unknown. The changes to the RBC membrane and anion transport across the membrane caused by SAO could impact development of malarial parasites in several ways. SAO may alter the ability of the parasite to develop within the erythrocyte. The altered membrane characteristics of SAO erythrocytes may impair the parasite's ability to remodel the RBC surface [53] and affect deformability of the infected erythrocytes resulting in impaired transit through capillaries, while the changes induced by the decrease in anion transport and gas exchange [54] might inhibit growth of parasites inside the SAO RBC. Alternatively, SAO may alter the ability of *P. vivax* to attach to and/or invade reticulocytes Although SAO reduced parasite densities even in infants aged ≤12 mo, this resulted in a reduced prevalence of infection only in the second year of life. This indicates that SAO cells may not be inherently resistant to *P. vivax* infection, but that children acquire this protective effect only in concert with increasing acquisition of anti-blood-stage immunity, either by increasing the acquisition and/or by enhancing the protective effect of immune response. A similar interaction between host genetics and age-specific immune status has recently be shown for the sickle cell trait (HbAS), where in cohort of Ugandan children the youngest children were best protected against high density parasitaemia, while only older children were protected against establishment of parasitaemia [55].

In contrast to normal RBCs where band 3 is mainly found as dimers, protein expressed from the *SLC4A1Δ27* allele may induce conformational changes in the normal band 3 protein resulting in the predominance of band 3 hetero-tetramers, higher order hetero-oligomers, and aggregates in SAO RBCs [56]. How this influences the distribution of other RBC membrane proteins is not known. The predominance of higher order band 3 aggregates in SAO RBCs could, however, have a significant impact on the interaction between parasite ligands and RBC proteins in general, and specifically on the invasion of *P. vivax* via the Duffy antigen that is thought to be part of a 4.1R-based macromolecular complex that contains band 3 as a dimer [42].

In our studies that further explored the relationship between SAO and the Duffy antigen, fluorescence activated cell sorting (FACS)-based analyses revealed that SAO and non-SAO red cells expressed similar amounts of surface-level Duffy antigen and that PvDBPII bound to SAO and non-SAO cells equally well. Interestingly, we found that while antibody levels to PvDBPII measured by ELISA did not differ, SAO children were 3.3 times more likely than non-SAO children to have high levels of PvDBPII-specific BIAbs. This preferential production of functional BIAbs could occur because critical binding regions of PvDBP might be exposed for a longer period of time during less efficient invasion of SAO cells, making the protein more accessible to the host immune response that generate broadly binding inhibitory antibodies. Adjusting for blocking antibodies did not, however, change the magnitude of the protection provided by SAO against reinfection with *P. vivax*. Thus, while SAO increases the likelihood that high activity Duffy blocking antibodies are acquired, the protection attributed to SAO is distinct from that provided by Duffy blocking antibodies. Further study of SAO-based protection against *P. vivax* illness, in particular in small children who suffer the highest morbidity burdens of *P. vivax* morbidity [9], is required to determine how this mutation imparts its selective advantage.

Irrespective of the mechanism by which this mutation evolved, our observations highlight the potential contribution of *P. vivax* malaria in shaping the unique RBC polymorphisms found in Asian and Pacific populations. Future studies on host genetic adaptation in the Asia Pacific region should thus not exclusively focus on *P. falciparum* but need to include other human malaria species.

## Supporting Information

Table S1 Baseline characteristics of SAO and non-SAO children in the infant cohort and association with other common RBC polymorphisms.
(DOCX)

Table S2 Baseline characteristics of SAO and non-SAO children 5–14 y of age in the treatment – reinfection cohort and associations with other common RBC polymorphisms.
(DOCX)

Text S1 SAO children with clinical signs and symptoms of *P. falciparum* cerebral malaria.
(DOCX)

## Abbreviations

aHR: adjusted hazard ratio
aOR: adjusted odds ratio
BIAb: binding inhibitory antibody
IQR: inter-quartile range
IRR: incidence rate ratio
LDR-FMA: ligase detection reaction-fluorescent microsphere assay
LM: light microscopy
OR: odds ratio
PvDBP: *P. vivax* Duffy binding protein
RBC: red blood cell
SAO: Southeast Asian ovalocytosis
SP: sulphadoxine/pyramethamine
