# Supplementary material for: Reduced Risk of Plasmodium vivax Malaria in Papua New Guinean Children with Southeast Asian Ovalocytosis in Two Cohorts and a Case-Control Study
Source: PLoS Med. 2012 Sep 4;9(9):e1001305. doi: 10.1371/journal.pmed.1001305 (PMC3433408; doi:10.1371/journal.pmed.1001305)
Supplement: Table S1 — Baseline characteristics of SAO and non-SAO children in the infant cohort and association with other common RBC polymorphisms. (DOCX) [file pmed.1001305.s001.docx]

**Table S1**: Baseline characteristics of SAO and non-SAO children in the infant cohort and association with other common red blood cell polymorphisms.

|  |  |  |  |  |  |  |  |  |  |
| --- | --- | --- | --- | --- | --- | --- | --- | --- | --- |
|  |  | **SAO (***wt/Δ27***)** | |  | **non-SAO** (*wt/wt*) | |  |  |  |
| **Covariates** |  | **(n=130)** | |  | **(n=991)** | |  | **p-value** |  |
|  |  |  |  |  |  |  |  |  |  |
|  |  |  |  |  |  |  |  |  |  |
| **Treatment allocation** |  |  |  |  |  |  |  |  |  |
| AQ-SP |  | 51 | 39% |  | 323 | 33% |  |  |  |
| ART-SP |  | 38 | 29% |  | 336 | 34% |  |  |  |
| Placebo |  | 41 | 32% |  | 332 | 34% |  | 0.30 |  |
|  |  |  |  |  |  |  |  |  |  |
| Gender (male) |  | 68 | 52% |  | 508 | 51% |  | 0.82 |  |
|  |  |  |  |  |  |  |  |  |  |
| Village of residence |  |  |  |  |  |  |  |  |  |
| Biranis |  | 5 | 4% |  | 51 | 5% |  |  |  |
| Megiar |  | 4 | 3% |  | 55 | 6% |  |  |  |
| Aronis (Garup, Wasabamal, Zizzi) |  | 13 | 10% |  | 105 | 11% |  |  |  |
| Basken / Dimer |  | 33 | 25% |  | 190 | 19% |  |  |  |
| Bunu / (Kusen) Mugil |  | 22 | 17% |  | 124 | 13% |  |  |  |
| Matukar |  | 7 | 5% |  | 86 | 9% |  |  |  |
| Dylup |  | 5 | 4% |  | 66 | 7% |  |  |  |
| Karukm |  | 5 | 4% |  | 39 | 4% |  |  |  |
| Mirap |  | 7 | 5% |  | 64 | 6% |  |  |  |
| Sareng |  | 9 | 7% |  | 65 | 7% |  |  |  |
| Talidig |  | 13 | 10% |  | 109 | 11% |  |  |  |
| Rempi |  | 7 | 5% |  | 37 | 4% |  | 0.55 |  |
|  |  |  |  |  |  |  |  |  |  |
| Season of enrolment - dry |  | 32 | 25% |  | 271 | 27 |  | 0.51 |  |
|  |  |  |  |  |  |  |  |  |  |
| Mean ITN use^1^ |  | 0.80 | [0.27] |  | 0.82 | [0.26] |  | 0.36 |  |
|  |  |  |  |  |  |  |  |  |  |
| **Common RBC polymorphisms** |  |  |  |  |  |  |  |  |  |
|  |  |  |  |  |  |  |  |  |  |
| *α^+^-thalassaemia* |  |  |  |  |  |  |  |  |  |
| αα/αα |  | 25 | 20% |  | 192 | 20% |  |  |  |
| αα/α- |  | 57 | 45% |  | 449 | 46% |  |  |  |
| α-/ α- |  | 46 | 36% |  | 343 | 35% |  | 0.96 |  |
|  |  |  |  |  |  |  |  |  |  |
| *Gerbich blood group* |  |  |  |  |  |  |  |  |  |
| wt/wt |  | 84 | 65% |  | 627 | 63% |  |  |  |
| wt/Δex3 |  | 36 | 28% |  | 283 | 29% |  |  |  |
| Δex3/Δex3 |  | 10 | 8% |  | 80 | 8% |  | 0.96 |  |
|  |  |  |  |  |  |  |  |  |  |

^1^ Average reported bednet use during the 7 study visits reported as mean and standard deviation
