# Supplementary material for: Reduced Risk of Plasmodium vivax Malaria in Papua New Guinean Children with Southeast Asian Ovalocytosis in Two Cohorts and a Case-Control Study
Source: PLoS Med. 2012 Sep 4;9(9):e1001305. doi: 10.1371/journal.pmed.1001305 (PMC3433408; doi:10.1371/journal.pmed.1001305)
Supplement: Table S2 — Baseline characteristics of SAO and non-SAO children 5–14 y of age in the treatment – reinfection cohort and associations with other common RBC polymorphisms. (DOCX) [file pmed.1001305.s002.docx]

**Table S2**: Baseline characteristics of SAO and non-SAO children 5-14 years of age in the treatment – reinfection cohort and associations with other common red blood cell polymorphisms

|  |  |  |  |  |  |  |  |  |  |
| --- | --- | --- | --- | --- | --- | --- | --- | --- | --- |
|  |  | **SAO (***wt/Δ27***)** | |  | **non-SAO** (*wt/wt*) | |  |  |  |
|  |  | (n=27) | |  | (n=179) | |  | p-value |  |
|  |  |  |  |  |  |  |  |  |  |
|  |  |  |  |  |  |  |  |  |  |
| **Baseline Characteristics** |  |  |  |  |  |  |  |  |  |
| Gender (male)^a^ |  | 12 | 44.4% |  | 88 | 49.2% |  | 0.65 |  |
|  |  |  |  |  |  |  |  |  |  |
| Age (mean) ^a^ |  | 9.5 | [8.9, 10.1] |  | 9.4 | [9.1, 9.6] |  | 0.71 |  |
|  |  |  |  |  |  |  |  |  |  |
| Enrolled at Mugil ES |  | 26 | 96.3% |  | 126 | 70.4% |  | 0.004 |  |
| Distance from School ^a^ |  | 1.34 | [1.01, 1.68] |  | 1.52 | [1.35, 1.69] |  | 0.34 |  |
| Distance from Cost ^a^ |  | 1.68 | [1.20, 2.15] |  | 1.55 | [1.36, 1.75] |  | 0.63 |  |
|  |  |  |  |  |  |  |  |  |  |
| Regular ITN user |  | 9 | 33.3% |  | 62 | 34.6% |  | 0.89 |  |
|  |  |  |  |  |  |  |  |  |  |
| mean Hb ^a^ |  | 10.8 | [10.2, 11.4] |  | 11.3 | [11.1, 11.5] |  | 0.14 |  |
| Enlarged spleen |  | 15 | 55.6% |  | 83 | 47.7% |  | 0.45 |  |
|  |  |  |  |  |  |  |  |  |  |
| ***Parasitology*** |  |  |  |  |  |  |  |  |  |
| (post PCR LDR-FMA) |  |  |  |  |  |  |  |  |  |
| *P. vivax* only |  | 4 | 14.8% |  | 21 | 11.7% |  |  |  |
| *P. falciparum* only |  | 15 | 55.6% |  | 79 | 44.1% |  |  |  |
| Mixed *Pf*/*Pv* infection |  | 3 | 11.1% |  | 42 | 23.5% |  | 0.44 |  |
|  |  |  |  |  |  |  |  |  |  |
| **Common RBC polymorphisms** |  |  |  |  |  |  |  |  |  |
|  |  |  |  |  |  |  |  |  |  |
| *α^+^-thalassaemia* |  |  |  |  |  |  |  |  |  |
| αα/αα |  | 5 |  |  | 26 |  |  |  |  |
| αα/α- |  | 11 | 40.7% |  | 82 | 39.7% |  |  |  |
| α-/ α- |  | 11 | 40.7% |  | 71 | 40.7% |  | 0.78 |  |
|  |  |  |  |  |  |  |  |  |  |
| *Gerbich blood group* |  |  |  |  |  |  |  |  |  |
|  |  | *19* |  |  | *133* |  |  |  |  |
| wt/Δex3 |  | 8 | 29.6% |  | 38 | 21.2% |  |  |  |
| Δex3/Δex3 |  | 0 | 0.0% |  | 8 | 4.5% |  | 0.47^b^ |  |
|  |  |  |  |  |  |  |  |  |  |

^a^ 95% confidence intervals estimated from Student’s t-test assuming equal variance.

^b^ Fisher’s exact test
