# Supplementary material for: Reduced Risk of Plasmodium vivax Malaria in Papua New Guinean Children with Southeast Asian Ovalocytosis in Two Cohorts and a Case-Control Study
Source: PLoS Med. 2012 Sep 4;9(9):e1001305. doi: 10.1371/journal.pmed.1001305 (PMC3433408; doi:10.1371/journal.pmed.1001305)
Supplement: Text S1 — SAO children with clinical signs and symptoms of P. falciparum cerebral malaria. (DOCX) [file pmed.1001305.s003.docx]

**SAO children with clinical signs and symptoms of *P. falciparum* cerebral malaria**

Of the 283 *P. falciparum* infected children that full WHO criteria for severe malaria, 16 (5.7%) were heterozygous for *SLC4A1Δ27* indicating the presence of SAO phenotype. Three of these children were deeply comatose as determined by the presence of a Blantyre Coma Score (BCS) less than 3, 30 minutes, 1 hour and 6 hours after correction of hypoglycaemia, the last seizure and anticonvulsant therapy, respectively.

Case 1: Female, aged 4.3yrs presented with BCS=2 and had evidence of *P. falciparum* malaria by light microscopy (LM) (parasitaemia count of 4266 parasites/µL), and *P. falciparum* infection confirmed by PCR and RDT. A full neurological examination revealed grinding teeth (bruxism) and up-going plantar responses, findings that are consistent previous case series of cerebral malaria from Papua New Guinea [[1](#_ENREF_1),[2](#_ENREF_2),[3](#_ENREF_3)]. Her haemoglobin (Hb) concentration was 9.8g/dl and lactate, glucose and bicarbonate concentrations were all within the normal range. Examination of cerebrospinal fluid (CSF) revealed normal protein, glucose and cell counts. Blood cultures were also negative. She was discharged with neurological deficits in motor function.

Case 2: Male, aged 3.2yrs presented with BCS=1, a scanty *P. falciparum* parasitaemia (219 parasites/µL) and *P. falciparum* infection confirmed by PCR and RDT. His Hb was 8.3 g/dl. He presented with prolonged, multiple seizures, decreased muscle tone and diminished reflexes. His CSF was normal.

Case 3: Female, aged 2.0yrs presented with BCS=2, a scanty *P. falciparum* parasitaemia (309 parasites/µL) and *P. falciparum* infection confirmed by both PCR and RDT). Her Hb was 6.1 g/dl. She presented following a single seizure and was treated with with paraldehyde. Neurological examination revealed decreased muscle tone and diminished reflexes. No CSF was collected.

Cases 2 and 3 had blood cultures taken and no significant bacterial pathogen was identified to account for the presenting illness. Lactate, glucose and bicarbonate concentrations were all within the normal range. Although every effort was made to standardise the recording of the BCS in relation to the timing of seizures and administration of anticonvulsants, both these features were prominent in the clinical presentations of cases 2 and 3. Although malaria remains the most likely aetiology in both, the possibility that a prolonged post-ictal phase after seizures, non-convulsive status epilepticus or prolonged effects of anticonvulsant therapy could contribute to the deep coma observed in these cases cannot be discounted.

The clinical, laboratory and prognostic features of all 3 children are summarized in Table I.

In addition, to these 3 children with deep coma, 3 further SAO children presented with impaired consciousness (BCS = 4). They were infected with *P. falciparum* by LM (parasitaemias of 15605/µL, 544/µL and 160/µL, respectively) and malaria infection was confirmed by the presence of *P. falciparum* DNA by PCR in the first 2 mixed *P.falciparum/P.vivax* DNA in the third. One SAO child presented with impaired consciousness and *P. vivax* infection (see main text for details).

In this study, we have documented one definite case and two possible cases of cerebral malaria caused by *P. falciparum* in children with SAO. This suggests that, in contrast to previous reports, SAO does not provide 100% protection against cerebral malaria in Papua New Guinean children.

1. Allen SJ, O'Donnell A, Alexander ND, Clegg JB (1996) Severe malaria in children in Papua New Guinea. QJM 89: 779-788.

2. Genton B, Al Yaman F, Mgone CS, Alexander N, Paniu MM, et al. (1995) Ovalocytosis and cerebral malaria. Nature 378: 564-565.

3. Allen SJ, O'Donnell A, Alexander ND, Mgone CS, Peto TE, et al. (1999) Prevention of cerebral malaria in children in Papua New Guinea by southeast Asian ovalocytosis band 3. AmJTropMedHyg 60: 1056-1060.

**Table I**. Clinical, laboratory and prognostic features of children with SAO genotype and cerebral malaria or

| Sex  /Age (y) | BCS | Parasit-aemia (parasites/µL) | PCR | RDT | Neurological signs | Cerebro-  spinal  fluid | Blood culture | Glucose  Lactate  Bi-carbonate | Outcome |
| --- | --- | --- | --- | --- | --- | --- | --- | --- | --- |
| F/4 | 2 | Pf 4266 | Pf | Pf | Short febrile seizure; upgoing plantar responses and bruxism | Normal | Negative | Normal | Discharged with deficit in motor function |
| M/3 | 1 | Pf 219 | Pf | Pf | Prolonged, multiple seizures; decreased muscle tone and reflexes | Normal | Negative | Normal | Discharged well |
| F/2 | 2 | Pf 309 | Pf | Pf | Single seizure; given paraldehyde, decreased muscle tone and reflexes | Not done | Negative | Normal; | Discharged well |

malaria with impaired consciousness

BCS; Blantyre coma score, F; female, M; male, Pf; *Plasmodium falciparum,*Pv; *Plasmodium vivax*
